# Remote Hemodynamic Monitoring in Heart Failure Management: A Comprehensive Review of Recent Advances and Clinical Challenges

**DOI:** 10.3390/biomedicines13112731

**Published:** 2025-11-07

**Authors:** Carmen M. Galvez-Sánchez, Julio A. Camacho-Ruiz, Lorys Castelli, Rosa M. Limiñana-Gras

**Affiliations:** 1 Department of Personality, Evaluation and Psychological Treatment, Faculty of Psychology and Speech Therapy, University of Murcia, Building 31, 30100 Murcia, Spain; julioangel.camachor@um.es (J.A.C.-R.); liminana@um.es (R.M.L.-G.); 2 Regional International Campus of Excellence (CEIR) Mare Nostrum Campus (CMN), 30100 Murcia, Spain; 3 Foundation Project Man Jaén, 23002 Jaén, Spain; 4 Department of Psychology, University of Turin, 10124 Turin, Italy; lorys.castelli@unito.it; 5 Assisted Reproduction Unit, QuironSalud Murcia Medical Center, 30008 Murcia, Spain

**Keywords:** hemodynamic monitoring, heart failure, HF, challenges, adherence

## Abstract

**Background/Objectives:** Heart Failure (HF) remains a leading cause of hospitalization and mortality worldwide, representing a significant burden on patients and healthcare systems. Despite advances in pharmacological and device-based therapies, readmission rates remain high and traditional monitoring approaches often fail to detect early physiological deterioration. This review examines the clinical utility and implementation challenges of remote hemodynamic monitoring in HF, highlighting its role in improving patient outcomes and guiding precision care. **Method:** A comprehensive narrative review was conducted using PubMed, Scopus, and Web of Science databases to identify peer-reviewed English-language studies published in the past ten years. **Results:** Monitoring hemodynamic status is essential for preventing HF readmissions, as elevated filling pressures often precede symptoms. Previous studies suggest that traditional methods may be less effective in detecting early changes, which could contribute to delays in initiating treatment. Remote monitoring offers continuous, individualized assessment and has shown potential to reduce hospitalizations, though its effectiveness varies across populations and settings. Telemonitoring primarily targets patients at higher risk of hospitalization, such as those classified as New York Heart Association(NYHA) class III and individuals with comorbidities that exacerbate HF. Remote hemodynamic monitoring presents notable clinical advantages, although its widespread adoption faces several challenges (i.e., the invasiveness of some monitoring systems; limited patient adherence due to technical complexity or cognitive and physical barriers; difficulties associated with comorbidities; variability in the efficacy of monitoring strategies across populations; difficulties faced by healthcare teams in managing and interpreting large volumes of real-time data; cost-effectiveness issues related to devices and infrastructure costs). Addressing these limitations will be essential to fully understanding the potential of remote monitoring in HF care. **Conclusions:** Remote hemodynamic monitoring enables early detection of physiological deterioration in HF, allowing timely interventions that reduce hospitalizations and improve outcomes. Emerging evidence suggests that, in contrast to traditional approaches, this method has the potential to support more personalized, data-driven care. Integrating biopsychosocial, gender, and intersectional perspectives further aligns this strategy with precision medicine, enhancing its effectiveness and equity in clinical practice. Despite promising recent advances, further research is essential to broaden the scientific evidence base and to enhance support for clinical decision-making.

## 1. Introduction

Heart failure (HF) is a complex clinical syndrome resulting from structural or functional impairments of the ventricles, ultimately leading to compromised systolic and/or diastolic performance and symptomatic left ventricular dysfunction. Clinically, it is primarily manifested through cardinal symptoms such as exertional dyspnea, fatigue, reduced exercise tolerance, and signs of fluid overload, including peripheral edema and pulmonary congestion [1].

Beyond its clinical complexity, HF represents a major global health challenge due to its high prevalence, significant morbidity and mortality, and recurrent hospitalizations. Epidemiologically, it imposes a substantial burden on healthcare systems, particularly in aging populations, and is associated with poor long-term prognosis despite advances in pharmacological and device-based therapies. The progressive nature of HF and its frequent exacerbations highlight the urgent need for innovative strategies aimed at early detection, close monitoring, and individualized management [2].

Cardiovascular diseases remain the leading cause of mortality worldwide, accounting for an estimated 17.9 million deaths in 2019. In Spain, more than 770,000 individuals are currently living with HF, with prevalence rates ranging from 4.7% to 6.8% among adults over 45 years of age. HF accounts for over 25% of all hospital admissions due to cardiovascular conditions, underscoring its substantial clinical and systemic impact. In-hospital mortality exceeds 10%, rising to nearly 20% within the first year after discharge and reaching 40–50% at five years [3].

From an economic perspective, HF entails a considerable burden on the Spanish healthcare system, with an estimated annual cost of approximately €2.5 billion, representing about 3.8% of total national health expenditure. Hospital-related costs alone surpass €470 million. These figures emphasize the need to strengthen coordination across levels of care, develop preventive strategies, promote healthy lifestyle behaviors, and allocate sufficient resources to support the accreditation and operation of specialized HF units. In this context, advancing the use of remote hemodynamic monitoring may offer a promising strategy to optimize disease management, reduce preventable hospitalizations, and improve clinical outcomes in patients with HF [3].

Hemodynamic monitoring has emerged as a valuable approach to reducing hospital readmissions by enabling early detection of decompensation and guiding proactive management. Its primary advantage lies in its ability to detect subclinical congestion. Techniques such as pulmonary artery pressure monitoring can identify elevations in cardiopulmonary pressures before the onset of overt symptoms, providing a crucial window for early therapeutic intervention. This facilitates timely optimization of treatment regimens, as hemodynamic data inform clinicians’ decisions regarding adjustments in pharmacologic therapy based on predefined pressure thresholds. By maintaining patients within target hemodynamic ranges, this approach mitigates HF progression and decreases the likelihood of acute exacerbations requiring hospitalization [4].

CardioMEMS is an FDA-approved remote hemodynamic monitoring system that uses a wireless sensor implanted in the pulmonary artery to measure hemodynamic pressures. This device enables patient management based on hemodynamic goals rather than symptom severity, allowing early therapeutic interventions and facilitating proactive care. Evidence from clinical trials, particularly the CHAMPION study, has demonstrated its effectiveness in reducing HF hospitalizations, showing a 28% reduction in admissions at six months and sustained benefits beyond two years. This protocol-driven model of care, focused on maintaining pulmonary artery pressures below high-risk thresholds, has been shown to prevent symptomatic decompensation and reduce the need for acute hospital-based interventions. The CardioMEMS system has proven effective in patients with both preserved and reduced ejection fractions, broadening its applicability across the HF population [5,6].

However, the system still faces challenges related to daily patient compliance, device cost, infrastructure requirements for large-scale monitoring, and the invasive nature of sensor implantation. Although the CardioMEMS HF system is the most extensively studied, other devices—such as the Cordella HF System, Chronicle Monitoring System, HeartPOD, and V-LAP—have also been evaluated in clinical trials, with preliminary findings suggesting promising clinical outcomes. Nonetheless, the majority of efficacy data to date derive from studies conducted with the CardioMEMS system [4,5,6,7,8,9,10].

Remote monitoring, particularly wireless hemodynamic monitoring, has therefore become a valuable tool in the comprehensive management of HF. By enabling early detection of physiological deterioration, these technologies support well-timed therapeutic adjustments, ultimately reducing hospitalizations and improving quality of life. Clinical trials have demonstrated notable benefits in selected populations; however, the heterogeneity of results underscores the relevance of linking remote device data to structured and effective clinical decision-making pathways. The utility of remote monitoring depends not only on technological capabilities but also on the ability to act upon the data in a timely and personalized manner [4,5,6,7,8,9,10].

Recent advances in cardiac device technology—characterized by increased miniaturization, improved automation, and enhanced usability—have positioned remote monitoring as a potentially indispensable component of modern HF care. This evolution has been accelerated by the expansion of telehealth and virtual care models, particularly during and after the COVID-19 pandemic. Looking ahead, emerging developments in artificial intelligence (AI), machine learning, and real-time data integration are expected to enhance the predictive power and clinical utility of remote monitoring systems, supporting more proactive and personalized HF management [11].

Despite these promising outcomes, broader implementation of hemodynamic monitoring remains limited by factors such as the invasive nature of certain systems, patient adherence challenges, and concerns regarding cost-effectiveness. Addressing these barriers is essential to fully realize the potential of hemodynamic-guided management in HF care [4,5,6].

Health equity is a fundamental consideration in the implementation of remote monitoring strategies. Disparities in access to healthcare—particularly among vulnerable populations such as older adults, individuals with disabilities, and those facing socioeconomic limitations—may restrict the potential benefits of these technologies. Therefore, it is crucial to design remote monitoring systems that are accessible, inclusive, and responsive to the needs of diverse patient populations, ensuring that all individuals with HF can equitably benefit from these innovations.

This comprehensive review analyzes the challenges and strategies of remote monitoring to reduce rehospitalizations in patients with HF, highlighting its effectiveness and clinical relevance. It also addresses critical gaps in the current understanding of remote hemodynamic monitoring in HF management. Specifically, it explores underexamined areas such as health equity, patient adherence challenges, and the integration of remote monitoring technologies into real-world clinical pathways. By synthesizing recent evidence, this review provides novel insights into the clinical utility, cost-effectiveness, and implementation challenges of remote hemodynamic monitoring, while emphasizing the relevance of biopsychosocial, gender-sensitive, and intersectional perspectives in advancing precision medicine. These contributions are intended to inform future research and support the development of more effective, equitable, and patient-centered models of HF care.

## 2. Materials and Methods

This study employed a comprehensive narrative review methodology to explore the current evidence on remote hemodynamic monitoring in the management of heart failure. A targeted search was conducted across PubMed, Scopus, and Web of Science databases, focusing on peer-reviewed articles published in English over the past ten years. To ensure comprehensiveness, illustrative examples from prior studies of substantial relevance were also considered.

### 2.1. Inclusion and Exclusion Criteria

Studies were included if they addressed remote hemodynamic monitoring technologies in heart failure patients, with a focus on randomized controlled trials, cohort studies, and cost-effectiveness analyses. Exclusion criteria included studies not available in English, those with unclear methodologies, or those that did not provide relevant data on clinical or economic outcomes.

### 2.2. Search Strategy

The search strategy was developed using Medical Subject Headings (MeSH) terms to enhance precision and consistency in identifying relevant literature. Key terms included "hemodynamic monitoring", "heart failure", "adherence", "clinical challenges", and "cost-effectiveness". Boolean operators were used to combine terms and refine the search results.

### 2.3. Study Selection Process

The initial screening of studies was performed based on titles and abstracts, followed by a full-text review of the selected articles. Two independent reviewers conducted the selection process to minimize bias and ensure the quality of the included studies. In cases of disagreement, a third reviewer was consulted to reach a consensus.

### 2.4. Quality Assessment

As this is a narrative review, a formal risk-of-bias assessment was not conducted. Instead, the selected studies were critically appraised to evaluate their methodological rigor, conceptual coherence, and clinical relevance. Particular attention was given to the clarity of study design, adequacy of sample size, validity of measurement instruments, and consistency of reported outcomes. Methodological limitations and potential sources of bias described by the original authors were also considered when synthesizing and interpreting the evidence.

### 2.5. Data Analysis

Data extracted from the selected studies were organized into thematic categories, including clinical efficacy, adherence, implementation challenges, and cost-effectiveness. Descriptive and comparative methods were employed to identify patterns and trends in the reviewed literature.

## 3. Results

The following section summarizes current evidence on the effectiveness and clinical relevance of remote hemodynamic monitoring in reducing rehospitalizations among patients with HF. It also outlines major challenges and knowledge gaps related to its clinical utility, cost-effectiveness, and real-world implementation, emphasizing the relevance of equity-oriented and patient-centered approaches in advancing HF care. 

### 3.1. The Role of Hemodynamic Monitoring in HF

HF is a complex and heterogeneous clinical syndrome that presents significant challenges in preventing hospital readmissions, largely due to its chronic progression, clinical variability, and high burden of comorbidities. The condition encompasses both reduced and preserved ejection fraction phenotypes, each with distinct pathophysiological mechanisms and therapeutic responses, further complicating individualized treatment strategies [12,13]. Patients with HF frequently present with multiple coexisting chronic conditions—such as chronic kidney disease, diabetes mellitus, and atrial fibrillation—which contribute to clinical instability and require the use of complex, often burdensome pharmacological treatments. Unlike acute cardiovascular events such as myocardial infarction, which are typically episodic in nature, HF requires continuous, long-term disease management and patient self-care. Following episodes of decompensation, patients often experience a progressive decline in functional capacity and quality of life, increasing their reliance on intensive healthcare resources. This downward clinical trajectory underscores the urgent need for integrated, proactive approaches that address not only the hemodynamic profile but also the broader biopsychosocial complexity of patients living with HF [12,13].

**Figure 1:**
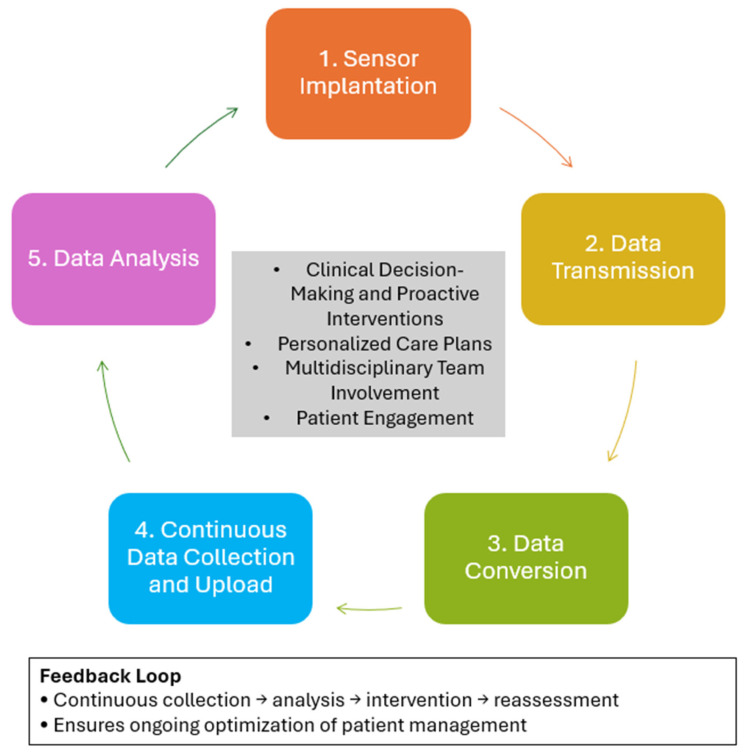
Workflow and Functional Principles of Remote Hemodynamic Monitoring Systems in Heart Failure.

Remote monitoring systems for HF (see Figure 1 for further details), such as the CardioMEMS HF system, operate through the continuous measurement of hemodynamic parameters—primarily pulmonary artery pressure (PAP) or left atrial pressure (LAP)—which reflect intracardiac filling pressures and fluid status. These systems consist of an implanted wireless sensor that transmits pressure data via an external antenna or reader to a secure, web-based management platform. The transmitted data are converted into pressure waveforms and related metrics, such as heart rate or activity level, providing clinicians with continuous, real-time information on patients’ hemodynamic status [14].

The collected data are automatically uploaded to cloud-based platforms, where algorithms analyze hemodynamic trends to detect early signs of deterioration. Some advanced systems also incorporate artificial intelligence to predict decompensation risk and provide actionable insights. Based on these analyses, healthcare providers can make timely pharmacological adjustments—such as modifying diuretic or vasodilator doses—before clinical symptoms worsen. This approach enables proactive and individualized management of HF patients [14].

Effective integration of remote monitoring into clinical care requires structured post-implantation protocols, collaboration among multidisciplinary teams (including cardiologists, nurses, and physician assistants), and active patient participation in daily monitoring routines. Continuous feedback between data collection, clinical interpretation, and therapeutic adjustment ensures that treatment plans remain responsive to each patient’s evolving hemodynamic profile [14].

Hemodynamic monitoring plays a crucial role in the clinical management of HF by providing direct and continuous assessment of cardiopulmonary congestion and informing individualized therapeutic strategies. Among its most significant contributions is the ability to measure intrathoracic pressures, particularly pulmonary artery pressures, which are tightly correlated with the pathophysiological progression of HF. These measurements facilitate the early detection of decompensation before the onset of overt symptoms, enabling timely intervention and preventing clinical deterioration. Through the continuous tracking of hemodynamic parameters, clinicians can tailor pharmacologic and non-pharmacologic treatments to maintain values within predefined safe thresholds, thereby reducing the incidence of hospital readmissions and acute episodes [4]. Remote hemodynamic monitoring, such as CardioMEMS, has been found to be cost-effective in optimizing care for chronic HF patients, improving quality of life, and reducing hospitalizations [15]. 

Among the available technologies for remote hemodynamic monitoring, the CardioMEMS HF system is the most extensively studied and validated device to date. Multiple randomized controlled trials, including CHAMPION, GUIDE-HF, and MONITOR-HF, have consistently demonstrated that the use of CardioMEMS is associated with significant reductions in HF-related hospitalizations. These trials reported hospitalization rate reductions ranging from 28% to 44% across diverse clinical settings and healthcare systems, supporting the generalizability of the findings. Furthermore, real-world observational studies have supported these results, with some reporting reductions in hospitalization rates as high as 80%, underscoring the device’s potential when integrated into standard care pathways [5,6,16,17].

In addition to clinical effectiveness, cost-effectiveness analyses—predominantly focused on the CardioMEMS system—have shown favorable incremental cost-effectiveness ratios (ICERs), typically remaining below the commonly accepted threshold of $50,000 per quality-adjusted life year (QALY) gained. These favorable economic outcomes are largely attributed to the significant reduction in hospitalizations, which offset the upfront costs related to device implantation, infrastructure, and ongoing monitoring. The main costs associated with this technology include the device itself, the implantation procedure, and the clinical time required for continuous patient follow-up and communication. However, the most substantial savings stem from the marked reduction in heart-failure-related hospitalizations (HFHs), which constitute a major component of overall healthcare expenditure due to prolonged admissions for decongestion. While most cost-effectiveness data are centered on CardioMEMS, emerging devices such as the Cordella HF System and the V-LAP monitoring system are currently under evaluation, with preliminary results indicating promising clinical and economic potential. Nevertheless, variations in healthcare systems and regional cost structures may influence implementation and overall economic outcomes [5,6,7,8,9,10].

Notable differences exist among clinical trials and monitoring devices. Most economic analyses are derived from data obtained in the CHAMPION trial, although more recent evaluations have incorporated findings from GUIDE-HF, COAST, and MEMS-HF. Other systems, such as the Cordella HF System, currently lack sufficient cost-effectiveness data because of limited regulatory approval and smaller available clinical datasets. As a result, comparative cost analyses across devices remain preliminary and should be interpreted with caution [6,7,8,9,10] (see Table 1 for further details).

Successful implementation of remote hemodynamic monitoring in routine HF care depends on several critical factors, including appropriate patient selection, integration within multidisciplinary care models, and the use of structured telemonitoring platforms. Patients with moderate to severe HF symptoms—particularly those classified as New York Heart Association (NYHA) class III—appear to derive the greatest benefit from these interventions. Overall, remote hemodynamic monitoring represents a transformative advancement in the management of HF, with the potential to improve clinical outcomes, enhance quality of life, and reduce the long-term burden on healthcare systems [14].

The temporal advantage offered by hemodynamic monitoring lies in its capacity to identify changes in pressure and volume status that precede symptomatic worsening. This preclinical time framework is critical for implementing interventions aimed at stabilizing the patient and preventing progression to more severe states of decompensation. The efficacy of remote hemodynamic monitoring systems—such as the CardioMEMS device—has been well established in clinical trials, where they have consistently demonstrated significant and sustained reductions in HF-related hospitalizations. These benefits have been observed across a broad spectrum of patients, including those with preserved and reduced ejection fraction, underscoring the wide applicability of this approach in diverse HF phenotypes [2,5,18]. Overall, while remote monitoring technologies improve HF management and reduce hospitalizations, their impact on all-cause mortality remains modest, emphasizing the need for further advancements in monitoring and therapeutic strategies [15].

Nonetheless, the implementation of hemodynamic monitoring is not without challenges. The invasive nature of device implantation, the need for consistent patient adherence to daily measurements, and the reliance on well-resourced healthcare infrastructure can limit its feasibility and scalability in routine clinical practice. Despite these limitations, hemodynamic monitoring remains the most effective remote monitoring strategy currently available for reducing HF readmissions. Continued technological innovation and integration with personalized care models are necessary to address existing barriers and enhance clinical utility [4].

For remote monitoring strategies to be clinically effective in HF, the parameters employed must meet several essential criteria. These include high accuracy in detecting true hemodynamic deterioration without being confounded by other physiological processes, as well as strong sensitivity and specificity to reliably identify early warning signs and avoid unnecessary interventions. Moreover, the ideal parameters should allow for early detection that provides sufficient time for therapeutic adjustment, and they must be easy to measure on a regular basis without causing discomfort or disrupting patients’ daily routines. Notably, these parameters must yield actionable insights that can directly inform and guide clinical decision-making to improve outcomes [4].

Several categories of monitoring parameters have been explored in this context. Hemodynamic variables, such as pulmonary artery pressure, offer a direct reflection of central congestion and are among the most precise indicators of volume status. Bioimpedance analysis has also been employed to estimate fluid accumulation and systemic congestion. Additionally, biomarkers such as natriuretic peptides provide biochemical insights into myocardial stress and fluid overload. Conventional measures, including body weight, blood pressure, and heart rate, along with patient-reported symptoms, continue to be used but are limited by low sensitivity and specificity, especially in the early stages of deterioration. Ultimately, the choice of monitoring parameters must strike a balance between diagnostic accuracy, level of invasiveness, and practical feasibility for both patients and healthcare systems [4].

Remote monitoring has emerged as a valuable tool in the management of HF, particularly for reducing hospitalizations associated with disease decompensation. One of its principal advantages lies in the early detection of changes in intracardiac filling pressures, which are key indicators of impending clinical deterioration. Unlike traditional monitoring approaches—such as symptom tracking or daily weight measurements—remote hemodynamic monitoring provides direct and continuous physiological data that reflect the underlying pathophysiology of HF. Devices such as the CardioMEMS system have demonstrated the ability to detect subtle increases in pulmonary artery pressure several weeks before the onset of symptoms or hospital admission, offering a critical window for timely therapeutic intervention [14].

By enabling daily assessments of hemodynamic status, remote monitoring supports a proactive, individualized approach to pharmacological management. Clinicians can make small, frequent adjustments to medications such as diuretics, nitrates, and renin–angiotensin–aldosterone system antagonists in response to real-time changes in pressure, thereby maintaining optimal ventricular filling and preventing decompensation. This level of precision allows for targeted interventions that go beyond reactive symptom management and contributes to improved clinical stability. Furthermore, remote monitoring enhances communication between patients and healthcare providers, fostering continuous engagement and rapid response to hemodynamic shifts. Together, these mechanisms illustrate how remote hemodynamic monitoring offers a path toward more dynamic, responsive, and personalized HF care [14].

### 3.2. Adherence to Telemonitoring in HF Patients

Patient adherence to telemonitoring interventions for HF management is influenced by a range of interrelated factors that behavioral aspects, technical, and systemic domains. One significant barrier is the complexity associated with self-monitoring routines. Many patients struggle to understand and consistently follow self-care instructions, which contributes to low rates of compliance. The usability of telemonitoring systems also plays a critical role; platforms that are perceived as difficult to operate or that require substantial effort may discourage regular use. Additionally, when telemonitoring disrupts patients established daily routines, adherence may be further compromised [4,13,19,20].

Association (NYHA) classification plays a fundamental role in identifying candidates for remote hemodynamic monitoring in HF. According to the evidence, patients classified as NYHA class III—those with moderate to severe HF—derive the greatest benefit from this approach. This is primarily because they are at higher risk of HF-related hospitalizations, particularly due to congestion, which represents the main therapeutic target of hemodynamic monitoring [14].

The CHAMPION and MONITOR-HF trials confirmed that patients in NYHA functional class III, who present significant symptoms and a history of hospitalizations for HF, experienced a substantial reduction in rehospitalizations following remote hemodynamic monitoring. In contrast, patients in lower NYHA classes with a reduced risk of hospitalization may benefit more from less intensive forms of telemonitoring [14,17].

Therefore, NYHA classification serves as a key criterion for identifying patients most likely to benefit from this technology, optimizing its use and improving cost-effectiveness in clinical practice [14].

Motivational and cognitive engagement are equally essential. A lack of awareness or understanding regarding the clinical relevance of telemonitoring can result in diminished patient participation. While ongoing support from healthcare providers—such as reminders or interactive follow-ups—has been proposed to enhance adherence, clinical trials have demonstrated only modest improvements using these strategies. Furthermore, technical limitations such as unreliable devices, intermittent connectivity, and data transmission failures can create feelings of frustration and hinder sustained use of telemonitoring systems [4,19,20].

Certain patient populations may also face intrinsic challenges to adherence. Individuals with physical disabilities or cognitive impairments often experience greater difficulties engaging with technology-dependent interventions. In addition, scepticism regarding the accuracy, relevance, and/or usefulness of the collected data may negatively influence trust in the system, reducing the likelihood of consistent use. Addressing these challenges through the development of user-friendly interfaces, comprehensive patient education, and accessible technical support is essential to improving adherence rates and maximizing the clinical impact of telemonitoring in HF care [4,19,20].

Interventions designed to reduce HF readmissions should focus on identifying barriers to the implementation of guideline-directed medical therapy (GDMT) and on developing comprehensive strategies to overcome them [13].

### 3.3. Challenges in Remote Monitoring for HF

Despite the potential of remote monitoring to transform HF management, several critical challenges must be addressed to improve its clinical utility, scalability, and overall impact. One of the primary obstacles lies in the selection of appropriate monitoring parameters. Ideal parameters should offer high accuracy and sensitivity while enabling the early detection of worsening HF without being confounded by unrelated physiological changes. However, identifying such parameters remains a significant research and clinical challenge [4].

As we previously analysed, patient’s adherence represents another major limitation. Compliance with self-monitoring strategies is frequently suboptimal, often due to the complexity of the systems, technological barriers, and the degree of disruption they pose to patients’ daily routines. Reported adherence rates can be as medium or low varying from 9 to 35%, severely compromising the effectiveness of even the most sophisticated monitoring technologies [4,19,20].

At the same time, healthcare providers face substantial challenges in managing the volume and complexity of transmitted data. Large datasets generated by remote systems require timely and accurate interpretation, and failure to act appropriately on these insights can diminish the intended benefits of early intervention. Additionally, several technological limitations persist. For example, bioimpedance-based monitoring typically depends on the presence of implantable devices, thereby restricting its use to a subset of patients. Although non-invasive alternatives are in development, they have yet to demonstrate equivalent efficacy or reliability [4].

Efficacy also remains a central concern. Many commonly used remote monitoring strategies, including daily weight tracking, biomarker surveillance, and impedance measurements, have shown limited effectiveness in reducing HF-related hospitalizations in large-scale clinical trials. In contrast, remote hemodynamic monitoring systems such as CardioMEMS have demonstrated clear benefits in this regard, but their use requires invasive implantation procedures, which may not be appropriate or acceptable for all patients [2,5,18].

Moreover, the cost-effectiveness of advanced remote monitoring technologies remains under scrutiny. Devices such as CardioMEMS demand significant infrastructure for widespread implementation and ongoing management, raising concerns about their scalability in real-world healthcare settings. Furthermore, these systems often rely on patient-initiated actions—such as triggering daily measurements—which introduces another potential point of failure, as non-adherence can undermine the benefits of monitoring and compromise clinical outcomes [2,5,18].

The successful implementation of remote hemodynamic monitoring in HF care requires more than technological advancement; it necessitates the structural integration of these systems into routine clinical workflows, along with well-defined protocols for patient selection and efficient strategies for data management and clinical response. As this review has demonstrated, remote hemodynamic monitoring holds considerable potential to transform the management of HF by enabling earlier detection of decompensation, facilitating personalized therapeutic adjustments, and ultimately reducing hospitalizations and improving patient outcomes. However, realizing these benefits on a broad scale depends on the ability of healthcare systems to embed remote monitoring into multidisciplinary care models, ensure equitable access, and promote adherence through patient-centered approaches. Moving forward, the integration of remote hemodynamic monitoring into personalized, proactive HF care represents a meaningful step toward improving quality of life and reducing the overall burden of the disease [14].

**Figure 2:**
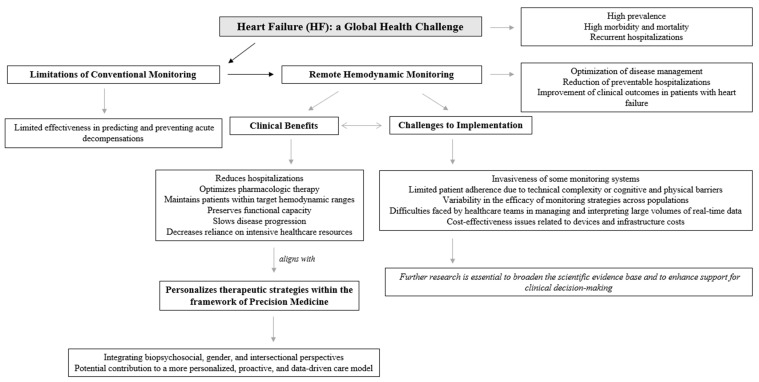
Remote hemodynamic monitoring in HF.

In sum, these challenges underscore the need for further innovation and refinement in remote monitoring strategies for HF. Future efforts should focus on developing more accessible, reliable, and patient-friendly technologies that offer clinically actionable data while minimizing invasiveness and cost. Reaching an optimal equilibrium among accuracy, accessibility, and scalability will be critical to translating the theoretical benefits of remote monitoring into tangible improvements in HF outcomes. Figure 2 provides a detailed conceptual map of remote hemodynamic monitoring in HF, derived from the present narrative review.

## 4. Discussion

The discussion is organized to provide a comprehensive synthesis of current evidence and emerging challenges in the use of remote hemodynamic monitoring for heart failure management. It sequentially examines the differences between remote hemodynamic and non-hemodynamic monitoring, clinical rationale and impact on readmission prevention, integration with pharmacological and device-based therapies, implementation challenges, and patient-specific determinants of response. Subsequent sections address geographical and economic differences, biopsychosocial and gender-sensitive perspectives, data accuracy issues, and future research priorities, offering an integrated framework for clinical translation and policy development.

### 4.1. Comparison Between Remote Hemodynamic and Non-Hemodynamic Monitoring

Remote hemodynamic monitoring provides distinct advantages over remote non-hemodynamic approaches in the management of HF. The primary differentiating factor lies in its capacity to directly assess intracardiac filling pressures. Hemodynamic systems, such as pulmonary artery pressure (PAP) sensors, can detect subclinical elevations in cardiac filling pressures several weeks before clinical decompensation, thereby enabling timely pharmacological adjustments and proactive management. In contrast, non-hemodynamic monitoring strategies—based on symptom reporting, body weight changes, or biomarker measurements—lack sensitivity and correlate poorly with intracardiac pressures, often failing to predict impending HF exacerbations [14].

Evidence from major randomized controlled trials (i.e., CHAMPION, GUIDE-HF, and MONITOR-HF) and real-world data consistently demonstrates that remote hemodynamic monitoring significantly reduces HF-related hospitalizations, whereas non-hemodynamic telemonitoring systems and cardiac implantable electronic devices (CIEDs) have shown limited or no benefit in this regard. Moreover, the hemodynamic approach promotes a proactive model of care by enabling early, pre-symptomatic interventions, while non-hemodynamic systems typically support reactive management based on late-stage clinical deterioration [14].

In summary, remote hemodynamic monitoring has proven superior in reducing hospitalizations and improving patient outcomes due to its ability to identify early hemodynamic changes and facilitate evidence-based, individualized treatment adjustments. Consequently, these systems have been endorsed by current HF management guidelines [14].

### 4.2. Clinical Rationale and Impact on Readmissions

Monitoring hemodynamic status and congestion plays a critical role in preventing hospital readmissions among patients with HF. Elevated intracardiac filling pressures, particularly in the pulmonary circulation, frequently precede the onset of clinical symptoms, providing a valuable opportunity for early detection and intervention before overt decompensation occurs. Traditional approaches based on symptom reporting or periodic assessments often fail to capture these early hemodynamic changes, resulting in delayed therapeutic action and increased risk of hospitalization.

In this context, remote hemodynamic monitoring technologies have emerged as a promising strategy to enable continuous assessment of congestion and guide timely, individualized management. Clinical evidence suggests that such systems can significantly reduce HF-related hospital admissions by facilitating early therapeutic adjustments based on objective physiological data rather than relying solely on patient-reported outcomes or physical examination findings [12,13].

Despite advances in pharmacological management and care coordination, reducing hospital readmissions remains a major challenge for the HF community. Several remote monitoring strategies have been introduced to decrease rehospitalization rates, yet their overall impact has been inconsistent across patient populations and clinical settings [4,15]. Differences in efficacy, limited patient access, technological complexity, and the financial burden associated with certain interventions—particularly those requiring invasive implantation or specialized infrastructure—represent significant barriers to widespread implementation [4].

Furthermore, disparities in digital literacy, adherence, and healthcare resource availability exacerbate these challenges, particularly among vulnerable or underserved populations. The development of more accessible, accurate, and cost-effective monitoring solutions that integrate physiological, psychological, and contextual data will be essential to overcoming these limitations. Future research should focus not only on technological innovation but also on health system integration, equity of access, and personalized care pathways. Ongoing progress in this area is indispensable for transforming remote monitoring from an emerging intervention into a clinically validated and widely implementable component of standard HF care [4].

### 4.3. Integration with Pharmacological and Device-Based Therapies

Remote hemodynamic monitoring can play a complementary role to pharmacological and device-based interventions by providing real-time, actionable data that supports individualized treatment adjustments and enhances clinical decision-making. Devices such as the CardioMEMS HF system can detect small increases in intracardiac filling pressures, including pulmonary artery or left atrial pressures, several weeks before HF-related hospitalizations occur. This early detection provides a critical window for timely pharmacological interventions, such as adjusting diuretic doses, thereby preventing decompensation and reducing the need for acute hospital care [17,21,22,23,24,25].

Hemodynamic monitoring also enables daily assessment of filling pressures, allowing for more frequent and personalized adjustments in medications, including diuretics, nitrates, renin–angiotensin–aldosterone system antagonists, and sympathetic nervous system antagonists. These individualized modifications help maintain optimal ventricular filling pressures, reduce congestion, and prevent excessive activation of compensatory neurohormonal systems. By guiding therapy based on direct hemodynamic data, clinicians can achieve a more stable physiological balance and improve overall clinical outcomes [17,21,22,23,24,25].

In addition, remote monitoring can be integrated with device-based therapies such as cardiac resynchronization therapy (CRT) or implantable cardioverter defibrillators (ICDs) [17,21,22,23,24,25]. Post hoc analyses have shown that combining remote hemodynamic monitoring with CRT reduces hospitalizations by enabling earlier detection of hemodynamic changes and facilitating more effective therapeutic adjustments [17,21,22,23,24,25]. The integration of remote monitoring data into secure, web-based platforms allows clinicians to analyze real-time trends and make evidence-based decisions. The use of algorithms and artificial intelligence might further enhance the interpretation of hemodynamic data, supporting timely and precise clinical decision-making [17,21,22,23,24,25].

Finally, remote monitoring promotes individualized and patient-centered care. Clinicians can define specific hemodynamic thresholds for each patient, adapting treatment decisions to individual needs and physiological responses. At the same time, patient engagement is strengthened through active participation in daily data collection and adherence to treatment recommendations, even during asymptomatic periods. This collaborative approach fosters improved adherence to pharmacological and lifestyle interventions, ultimately bridging the gap between medical therapy, device-based treatment, and real-world disease management.

### 4.4. Implementation Challenges and Training Needs

Reducing hospital readmissions in HF remains a persistent and multifaceted clinical challenge. This difficulty stems largely from the progressive and relapsing nature of the disease, as well as the limited capacity to fully prevent recurrent decompensations. HF is marked by high rates of early readmission following hospitalization, often within 30 days of discharge, and is associated with a steady decline in functional status and quality of life. The transition from stable chronic HF to acute decompensated HF is particularly concerning, as it dramatically increases the risk of adverse outcomes. Epidemiological data indicate that all-cause mortality rises from 6.4% to 23.6% within one year of an acute decompensation episode, reflecting the severity and fragility of this clinical trajectory [12,26,27,28].

Recent analyses have also challenged the assumption that readmissions are largely preventable. Estimates suggest that fewer than 25% of HF readmissions can be avoided through current interventions, highlighting the need to recalibrate expectations and strategies in managing post-discharge care. Moreover, although initiatives aimed at reducing 30-day readmission rates have yielded modest improvements in hospitalization metrics, they have coincided with a paradoxical 1.3% increase in short-term mortality. This unintended consequence raises important concerns about the validity of re-admission-based performance benchmarks and underscores the need for more nuanced, patient-centered outcome measures that capture the complexity of disease progression and care transitions in HF [14,26,27,28].

These challenges underscore the necessity of strategic planning, targeted training, and appropriate resource allocation to support the successful integration of remote monitoring technologies into routine clinical practice. The adoption of such systems requires not only technological acquisition but also a fundamental reconfiguration of existing care pathways. This includes the development of clear implementation protocols, the designation of clinical responsibilities within multidisciplinary teams, and the establishment of infrastructure for real-time data analysis and timely clinical decision-making. Additionally, healthcare professionals must receive adequate training to interpret hemodynamic data, adjust treatment regimens accordingly, and engage patients in the monitoring process to ensure adherence and optimize outcomes. From a systems perspective, adequate funding, institutional support, and alignment with broader health policy goals are essential to ensure long-term sustainability. Without addressing these organizational and operational dimensions, the clinical benefits demonstrated in controlled trials may not be fully achieved in real-world settings [14,29].

### 4.5. Clinical and Phenotypic Determinants of Response

Patient selection is a crucial determinant for the successful implementation of remote hemodynamic monitoring. Several HF phenotypes have been evaluated in this context, demonstrating variable but consistent clinical benefits across groups. Remote monitoring has proven effective in patients with both reduced (HFrEF) and preserved (HFpEF) ejection fraction, significantly decreasing HF-related hospitalizations despite differences in disease mechanisms [14].

The New York Heart Association (NYHA) classification also plays a fundamental role in identifying candidates most likely to benefit from this technology. Evidence indicates that patients in NYHA class III, characterized by moderate to severe HF, are at the highest risk of hospitalization due to congestion, the primary target of hemodynamic monitoring. The CHAMPION and MONITOR-HF trials demonstrated that these patients experienced a marked reduction in rehospitalizations through remote hemodynamic monitoring, whereas those with lower NYHA classifications may benefit more from less intensive telemonitoring modalities [14,17].

Beyond cardiac phenotype and functional class, comorbidities also play a significant role. Remote pulmonary artery pressure monitoring has shown benefits in HF patients with pulmonary hypertension, chronic obstructive pulmonary disease (COPD), chronic kidney disease, and obesity, as well as among those with CRT devices [14]. These findings highlight that remote monitoring is effective across a wide range of comorbidities, reinforcing its clinical relevance in high-risk populations [14].

### 4.6. Geographical and Economic Considerations

Geographical differences have also been observed in the context of remote hemodynamic monitoring, particularly regarding studies conducted across different regions and healthcare systems. The CHAMPION and GUIDE-HF trials were carried out in North America, whereas the MONITOR-HF trial was conducted in a European setting. Despite variations in study design and healthcare context, all demonstrated consistent reductions in HF-related hospitalizations [6,7,8,9,10,14].

Significant differences exist between European and U.S. healthcare systems in terms of costs, care protocols, and access to medical technologies. Cost-effectiveness analyses performed from a European perspective (including the United Kingdom, the Netherlands, Belgium, Italy, and Germany) revealed variations in expenses associated with hospitalization for HF, device implantation, and monthly monitoring, all of which influence the overall cost-effectiveness outcomes in each country. Although both European and North American studies have shown comparable clinical results, these differences in healthcare structures and economic frameworks may limit the generalizability of findings across regions. Nevertheless support consistent reduction in hospitalization rates observed in multiple contexts confirm the clinical relevance of remote hemodynamic monitoring in HF management [5,6,14,17]. 

Beyond these structural and economic contrasts, it is also essential to consider the geographical and sociodemographic characteristics of each region when interpreting and implementing these findings. Factors such as population density, healthcare infrastructure, digital connectivity, and socioeconomic inequalities can strongly influence both patient access and the feasibility of remote hemodynamic monitoring programs. Therefore, achieving equitable access to advanced cardiac devices and remote monitoring technologies represents a crucial goal to ensure that the clinical benefits demonstrated in controlled trials can be translated into real-world practice across diverse healthcare settings.

### 4.7. Biopsychosocial, Social Support, and Gender-Sensitive Perspectives

Given the multifactorial nature of HF and the complexity of remote monitoring, a comprehensive biopsychosocial approach is increasingly needed. While current strategies emphasize physiological parameters, long-term adherence and clinical success are also shaped by behavioral, psychological, and social factors. Patient engagement, emotional responses to chronic disease management, and the presence of supportive healthcare and social networks play a critical role in the effective use of monitoring technologies. Integrating these dimensions into HF care enhances adherence, aligns with personalized medicine, and improves outcomes [30].

Social support represents a critical yet often underappreciated determinant of adherence to therapeutic interventions. Individuals who receive consistent emotional, informational, and practical support from family, caregivers, and/or healthcare providers are more likely to engage with monitoring protocols and respond appropriately to therapeutic adjustments. Conversely, social isolation and limited caregiver involvement are associated with lower adherence and poorer outcomes. Structured support strategies—such as caregiver education, community-based programs, and peer-support networks—might therefore enhance engagement and adherence [31,32,33].

In addition, incorporating a gender-sensitive and intersectional approach [34] is essential for advancing equity and effectiveness. Evidence increasingly indicates that sex and gender differences influence HF epidemiology, access to care, engagement with health technologies, and clinical outcomes. Women remain underrepresented in trials and may present distinct symptom profiles and treatment responses. Intersectional factors—such as age, socioeconomic status, ethnicity, and caregiving responsibilities—further shape experiences with HF and remote monitoring. Ignoring these aspects risks reinforcing disparities and limiting generalizability. Therefore, hemodynamic monitoring of HF must systematically consider gender and intersectionality to ensure inclusivity and equity.

### 4.8. Risks, Data Accuracy, and False Alarms

Remote monitoring systems face several challenges related to adherence, sensitivity, invasiveness, and cost. Some systems, such as CardioMEMS, require invasive sensor implantation, which may not be suitable for all patients. Moreover, patient adherence remains a major limitation, as many individuals struggle with daily self-monitoring due to routine complexity or low digital literacy [35,36]. Technical issues—such as connectivity failures or unreliable sensors—can reduce patient trust and engagement. Healthcare providers also face challenges in managing large volumes of real-time data, and delayed responses may diminish early intervention benefits [2,4,5,18,19,20,37].

Another critical concern is the accuracy and reliability of generated data. False alarms or measurement errors can lead to inappropriate interventions or missed detections, compromising patient safety, increasing workload, and eroding trust in monitoring systems [4,19,20]. False alerts may also induce anxiety among patients and cause professional overload for clinicians. To mitigate these risks, monitoring systems must achieve high accuracy, sensitivity, and specificity, supported by structured decision-making protocols that ensure timely and appropriate clinical responses.

### 4.9. Future Directions and Policy Implications

Future research should aim to personalize and optimize remote hemodynamic monitoring by comparing outcomes between HF phenotypes, developing predictive algorithms integrating comorbidities, and exploring behavioral and socioeconomic determinants of adherence. Economic analyses are also needed to evaluate cost-effectiveness across diverse healthcare contexts and clinical complexities [14]. Preventive strategies, AI-driven analytics, patient education, and less invasive devices could reduce costs while improving engagement and outcomes. Nevertheless, further research is needed to ensure the responsible and ethical use of AI in this context, particularly regarding data privacy, algorithmic bias, and clinical decision-making transparency. Developing clear regulatory and ethical frameworks will also be essential to guarantee that these technologies enhance equity and patient-centered care rather than exacerbate existing disparities.

Moreover, integrating remote monitoring into standardized, multidisciplinary care pathways will be essential to determine effectiveness across broader populations. Large-scale pragmatic trials should assess long-term effects on quality of life, functional capacity, and resource use. Multimodal approaches combining telemonitoring with rehabilitation and psychological support could further enhance adherence and predictive power.

Finally, to strengthen external validity and ensure global applicability, future studies must include greater geographical and sociodemographic diversity. Comparative analyses of healthcare systems, reimbursement policies, and regional resource allocation will be vital for understanding implementation feasibility. In addition, policymakers’ perspectives should be incorporated to design equitable, context-sensitive, and sustainable models of remote HF care.

## 5. Conclusions

Remote hemodynamic monitoring has emerged as a promising strategy to reduce hospitalizations related to HF and improve overall patient outcomes. Despite significant advances in pharmacological and device-based therapies, conventional approaches to HF management remain limited in predicting and preventing acute decompensations. Traditional monitoring methods, often reliant on symptoms and periodic evaluations, frequently fail to identify early hemodynamic deterioration, leading to missed opportunities for timely intervention. In contrast, remote hemodynamic monitoring offers a proactive model of care by detecting subtle increases in intracardiac filling pressures, often weeks before the onset of clinical symptoms that typically prompt hospitalization. This early physiological insight enables clinicians to initiate tailored pharmacological adjustments and optimize volume status before patients reach a critical point of decompensation.

HF is a heterogeneous condition that manifests differently across patients, depending on comorbidities and disease phenotypes, including preserved or reduced ejection fraction. These variations influence both disease progression and therapeutic response. In this context, remote hemodynamic monitoring serves as a key instrument for implementing personalized management, enabling continuous assessment of physiological parameters and early detection of clinical deterioration. By integrating data on comorbidities and individual characteristics, telemonitoring systems can support more precise and timely therapeutic adjustments, optimizing treatment and enhancing quality of life. Moreover, comorbidities and social support play a crucial role in shaping adherence and the overall effectiveness of remote monitoring. Patients with multiple chronic conditions or limited social resources often face greater barriers to consistent engagement, underscoring the need to incorporate social context and multimorbidity into patient stratification and telemonitoring strategies. Thus, remote monitoring represents a major advancement in the transition toward anticipatory, individualized, and data-informed HF management.

Integrating biopsychosocial, gender-sensitive, and intersectional perspectives into remote hemodynamic monitoring strategies aligns closely with the principles of precision medicine. This approach tailors interventions to the unique biological, psychological, and social characteristics of each patient, moving beyond the traditional “one-size-fits-all” model. By considering not only hemodynamic and physiological parameters but also behavioral patterns, gender-related differences, and socio-contextual factors, clinicians can more accurately predict risk, personalize therapeutic targets, and improve adherence to monitoring technologies.

To sum up, future cost-effectiveness evaluations should also account for regional variability in healthcare infrastructure, reimbursement mechanisms, and resource allocation, as these factors critically determine the scalability and sustainability of remote monitoring systems across diverse health settings. Such multidimensional profiling enhances the capacity to deliver interventions that are both clinically effective and contextually relevant, advancing a truly patient-centered model of care. When embedded within a precision framework, remote monitoring technologies offer a powerful platform for dynamic, individualized, and equitable HF management. Although recent progress has introduced promising alternatives, further research remains essential to strengthen the evidence base and guide clinical decision-making in this field.

## Figures and Tables

**Table 1:** Summary of Main Clinical Trials Evaluating Remote Hemodynamic Monitoring Systems in HF.

Aspect	Description
Main Clinical Trials	The three most relevant clinical trials evaluating the CardioMEMS HF system are: CHAMPION (2011, North America), GUIDE-HF (2021, North America), and MONITOR-HF (2023, Europe).
Main Results	CHAMPION: 37% reduction in HF-related hospitalizations (HR: 0.63). GUIDE-HF: 28% reduction in hospitalizations in the pre-COVID-19 analysis (HR: 0.72). MONITOR-HF: 44% reduction in hospitalizations (HR: 0.56) and improved quality of life (KCCQ).
Differences in Inclusion Criteria	CHAMPION and MONITOR-HF included patients with moderate-to-severe HF (NYHA Class III) and a history of HF hospitalization within the previous 12 months. GUIDE-HF included patients with less advanced HF (NYHA II–IV) and elevated natriuretic peptide levels, even without prior hospitalization.
Geographical Context	CHAMPION and GUIDE-HF were conducted in North America, while MONITOR-HF was the first clinical trial carried out in a European setting. Despite differences in healthcare systems and trial conditions, the results consistently showed a reduction in hospitalizations.
Impact of the COVID-19 Pandemic	In GUIDE-HF, the overall analysis did not demonstrate a significant reduction in hospitalizations due to an interaction effect with the COVID-19 pandemic; however, the pre-COVID-19 analysis showed a clear reduction.
Comparison of Results	Summary of reductions in HF-related hospitalizations across studies: CHAMPION: 37% reduction. GUIDE-HF: 28% reduction (pre-COVID-19). MONITOR-HF: 44% reduction. MEMS-HF: 62% reduction. COAST: 82% reduction (real-world study). SIRONA-II: 73% reduction (Cordella HF System).
Differences Among Devices	Although the CardioMEMS HF system is the most extensively studied, other devices—such as the Cordella HF System, Chronicle Monitoring System, HeartPOD, and V-LAP—have also been evaluated in clinical trials. Most efficacy data, however, originates from studies using the CardioMEMS system.
Conclusions	Clinical trials have consistently demonstrated that remote hemodynamic monitoring reduces HF-related hospitalizations. Despite differences in inclusion criteria, geographic contexts, and specific outcomes, the benefits of this technology are evident, particularly in patients with moderate-to-severe HF.

**Abbreviations:** HF: heart failure; HR: hazard ratio; KCCQ: Kansas City Cardiomyopathy Questionnaire; NYHA: New York Heart Association.

## Data Availability

No new data were created or analyzed in this study. Data sharing is not applicable to this article.
